# Remote Adolescent-Onset Chorea Preceding Thrombotic Antiphospholipid Syndrome: A Case Report

**DOI:** 10.7759/cureus.114418

**Published:** 2026-08-12

**Authors:** Julian Andrés Gutiérrez Baquero, María Alejandra Prieto Zambrano, Juan Carlos Garzón Sutachán

**Affiliations:** 1 Neurology, Hospital Departamental de Villavicencio, Villavicencio, COL; 2 General Practice, Centro de Estudios Neurológicos de Villavicencio, Villavicencio, COL; 3 General Practice, Hospital Departamental de Villavicencio, Villavicencio, COL

**Keywords:** anticardiolipin antibodies, antiphospholipid syndrome, chorea, ischemic stroke, young adult stroke

## Abstract

Antiphospholipid syndrome (APS) is a systemic autoimmune disease characterized by vascular thrombosis and/or obstetric morbidity in the presence of persistent antiphospholipid antibodies. Chorea is an uncommon neurological manifestation, reported in approximately 1% of patients, and is not included among the clinical domains required for classification.

We report the case of a 28-year-old woman who presented with acute left-sided sensorimotor deficits. Her only relevant history was an episode of chorea involving the left upper limb at 18 years of age, which resolved spontaneously after an incomplete etiological workup at another institution. Neuroimaging demonstrated a right frontal ischemic lesion, and cardioembolic and large-vessel causes were excluded. Prompted by the prior history of chorea, testing revealed persistently high-titer anticardiolipin IgG and anti-β2-glycoprotein I IgG antibodies, establishing a diagnosis of primary APS. She was started on warfarin and achieved functional independence without recurrent vascular events.

This case illustrates that juvenile-onset chorea may represent the heralding manifestation of APS, preceding the qualifying thrombotic event by a decade. An isolated, self-limited chorea of undetermined cause should prompt antiphospholipid antibody testing, as early recognition may allow timely thromboprophylaxis and potentially prevent cerebrovascular events in young patients.

## Introduction

Antiphospholipid syndrome (APS) is a systemic autoimmune disorder defined by vascular thrombosis and/or obstetric manifestations occurring in the presence of persistently positive antiphospholipid antibodies (aPL) [1]. Its neurological spectrum extends well beyond ischemic stroke and includes cerebral venous sinus thrombosis, cognitive impairment, seizures, headache, transverse myelitis, and movement disorders [2].

Chorea is among the least frequent of these manifestations, reported in only 1.3% of patients in a Euro-Phospholipid cohort of 1,000 individuals with APS [3]. It is also a non-criteria manifestation: it does not appear among the six clinical domains of the 2023 ACR/EULAR (American College of Rheumatology/European Alliance of Associations for Rheumatology) classification criteria, so an isolated choreic episode cannot by itself contribute to classification, however suggestive it may be [4]. As a consequence, chorea arising in a young patient without an evident cause may be attributed to a self-limited process and its etiological workup left incomplete.

We report the case of a woman who developed isolated chorea at 18 years of age, for which the available workup was reportedly unrevealing but incomplete, and who, 10 years later, presented with an ischemic stroke that prompted antiphospholipid antibody testing and led to a diagnosis of primary APS. Because aPL were not measured at the time of the choreic episode, a causal relationship cannot be established. The case is therefore presented as an illustration of chorea as a possible antecedent non-criteria manifestation, and of the diagnostic opportunity that may be lost when otherwise unexplained chorea in a young patient is not fully investigated.

## Case presentation

A 28-year-old woman presented to the emergency department with a 3-day history of paresthesia and hypoesthesia of the left side of the face, associated with decreased muscle strength in the left hemibody and rightward deviation of the oral commissure.

Her only relevant personal history was an episode of chorea at 18 years of age. The movements were described by the assessing neurologist as involuntary, dance-like movements of the left upper limb that moved away from the midline. The episode was unilateral, occurred only once, resolved spontaneously within one month, and never recurred. No facial involvement, hypotonia, motor impersistence, or psychiatric symptoms were documented, and information on suppressibility and on proximal or distal predominance of the movements was not recorded. The patient was not taking oral contraceptives at that time. The external records indicate that brain imaging, antistreptolysin O titers, and serum copper and ceruloplasmin were requested, but the corresponding results were not available for review, and no autoimmune panel, antiphospholipid antibody testing, or cerebrospinal fluid analysis was documented. The workup performed at that time is therefore best characterized as reportedly unrevealing but incomplete.

On admission for the current event, the National Institutes of Health Stroke Scale score was 6. Examination documented left hemiparesis graded 4/5 on the Medical Research Council scale, hypoesthesia in a facio-brachio-crural distribution, and a left Babinski sign. The left biceps, brachioradialis, patellar, and Achilles reflexes were reduced; this finding was transient and normalized over the course of rehabilitation, a pattern consistent with the acute phase of an upper motor neuron lesion. She was not taking oral contraceptives and had no conventional cardiovascular risk factors.

**Figure 1:**
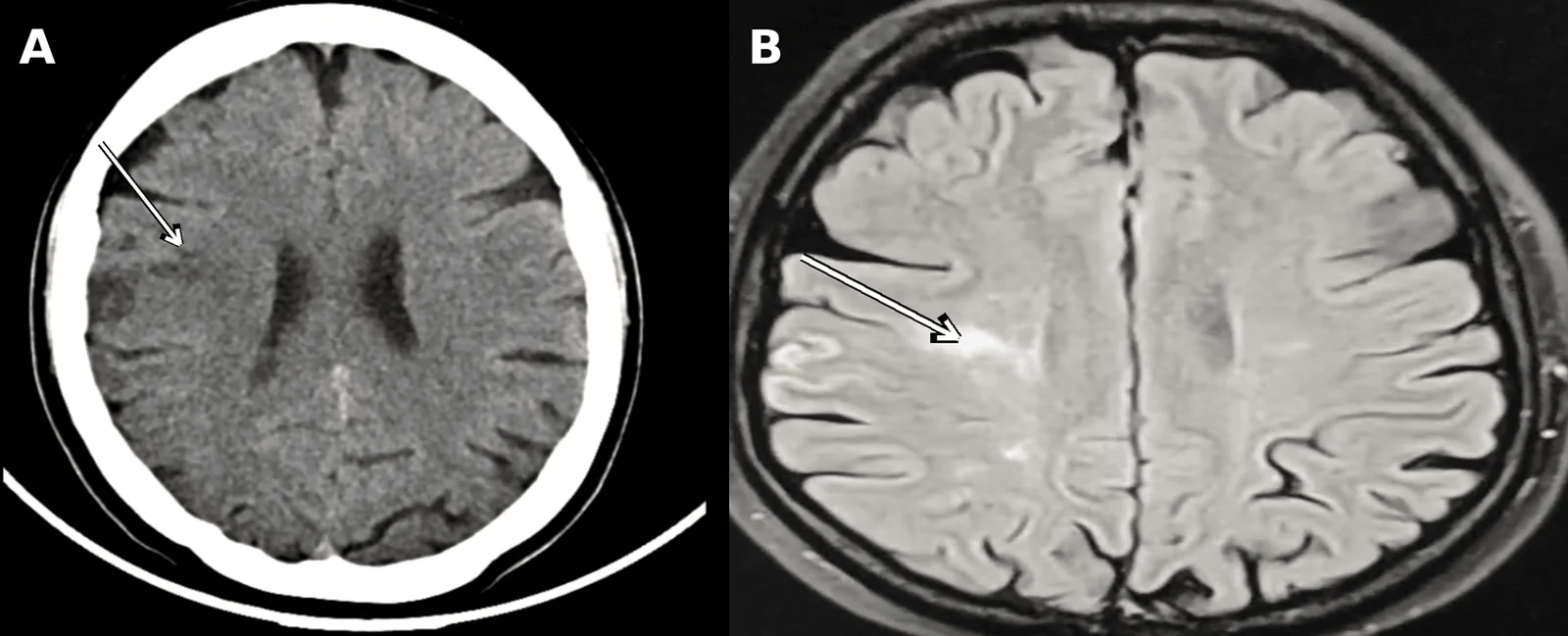
Neuroimaging findings at presentation (A) Non-contrast axial head CT obtained on day 1 after symptom onset, showing a subtle hypodense area in the right frontal white matter (arrow). (B) Contrast-enhanced axial fluid-attenuated inversion recovery MRI obtained on day 6, showing a corresponding area of increased signal in the posterior right frontal region with cortical involvement and extension into the adjacent deep white matter (arrow). The formal radiological report additionally documented gyriform enhancement and restricted diffusion, consistent with a subacute ischemic infarct.

Initial laboratory studies-- complete blood count, serum electrolytes, human immunodeficiency virus (HIV) antibodies, glycated hemoglobin, non-treponemal serology, and lipid profile--were within normal limits. Non-contrast head computed tomography (CT) performed on day 1 after symptom onset showed a subtle hypodense area in the right frontal white matter (Figure 1A). Contrast-enhanced brain magnetic resonance imaging (MRI) performed on day 6 defined the full extent of the lesion, demonstrating small areas of increased T2 signal with cortical involvement in the posterior region of the right frontal lobe and in the right parietal lobe, gyriform enhancement after contrast administration, and restricted diffusion, together with a band-like area of increased signal extending into the adjacent deep white matter and scattered subcortical T2 hyperintensities (Figure 1B). The combination of restricted diffusion and gyriform enhancement was consistent with a subacute ischemic infarct.

Given the clinical and imaging features, the presentation was classified as an ischemic stroke in a young adult, prompting an extended etiological workup for uncommon causes. CT angiography of the brain and neck vessels showed no vascular abnormalities, although cervical artery dissection was not specifically commented upon in the report. Transthoracic and transesophageal echocardiography, including an agitated saline study, showed no valvular disease, intracavitary thrombi, contractility abnormalities, or right-to-left shunt. Twenty-four-hour Holter monitoring detected no rhythm disturbances; inpatient telemetry was not performed, so paroxysmal atrial fibrillation cannot be entirely excluded. Inherited thrombophilia screening was not performed, in keeping with the limited role of such testing in arterial ischemic stroke.

Because the patient reported a history of chorea documented in external medical records but with an incomplete etiological workup at the time, the evaluation was extended to include an autoimmune panel and antiphospholipid antibodies (Table 1). Anticardiolipin IgG and anti-β2-glycoprotein I IgG antibodies were positive at high titers, defined by the classification criteria as values of 80 units or above measured by enzyme-linked immunosorbent assay, while the corresponding IgM isotypes were negative. The lupus anticoagulant, measured by the dilute Russell’s viper venom time before administration of heparin or any other anticoagulant, was negative. Both solid-phase assays remained positive at high titers on repeat testing 13 weeks later, confirming persistence.

**Table 1:** Antiphospholipid antibody profile at initial and confirmatory testing, autoimmune panel, and vasculitis workup ANCA: antineutrophil cytoplasmic antibodies; dRVVT: dilute Russell’s viper venom time; RU: relative units Methods: Enzyme-linked immunosorbent assay for anticardiolipin, anti-β2-glycoprotein I, and extractable nuclear antigen antibodies; dilute Russell's viper venom time for lupus anticoagulant; indirect immunofluorescence on HEp-2 cells for antinuclear antibodies; indirect immunofluorescence for anti-double-stranded DNA antibodies and ANCA, for which the substrate was not specified in the laboratory reports; immunoturbidimetry for C3 and C4; fluorescence enzyme immunoassay for anti-myeloperoxidase and anti-proteinase 3 Antiphospholipid antibodies, the autoimmune panel, and complement levels were obtained on admission; the vasculitis workup was performed one week later. Anticardiolipin and anti-β2-glycoprotein I antibodies were repeated 13 weeks later, confirming persistent positivity. The lupus anticoagulant was measured before administration of heparin or any other anticoagulant. Dashes indicate tests that were not repeated. The classification criteria define moderate (40-79 units) and high (≥80 units) titers for anticardiolipin and anti-β2-glycoprotein I antibodies measured by enzyme-linked immunosorbent assay; both IgG assays in this patient exceeded the high-titer threshold. The reporting laboratory provided the assay methodology and positivity cut-offs but not the manufacturer, analytical platform, or laboratory-specific 99th-percentile thresholds.

Test	Initial	Confirmatory	Reference range	Interpretation
Quantitative results				
Anticardiolipin IgG, U IgG-FL/mL	>120	>120	<12	Persistently positive (high titer)
Anticardiolipin IgM, U IgM-FL/mL	<2.00	<2.00	<12	Negative
Anti-β2-glycoprotein I IgG, RU/mL	157.37	150	<20	Persistently positive (high titer)
Anti-β2-glycoprotein I IgM, RU/mL	8.69	7.42	<20	Negative
Lupus anticoagulant, dRVVT ratio	1	Not repeated	<1.2	Negative
Anti-Sm, RU/mL	<2.00	—	<20	Negative
Anti-Ro/SS-A, RU/mL	<2.00	—	<20	Negative
Anti-La/SS-B, RU/mL	<2.00	—	<20	Negative
Anti-RNP, RU/mL	<2.00	—	<20	Negative
Anti-myeloperoxidase, IU/mL	0.3	—	<3.5	Negative
Anti-proteinase 3, IU/mL	<0.6	—	<2.0	Negative
Complement C3, mg/dL	105	—	83-193	Normal
Complement C4, mg/dL	17.4	—	15-57	Normal
Qualitative results				
Antinuclear antibodies	Negative (AC-0 pattern)	—	Positive: ≥1:160	Negative
Anti-double-stranded DNA IgG	Negative	—	Positive: ≥1:10	Negative
ANCA	Negative	—	No c-ANCA/p-ANCA fluorescence	Negative

Applying the 2023 ACR/EULAR classification criteria, the patient scored 4 points in the clinical domain (macrovascular arterial thrombosis in the absence of a high-risk cardiovascular profile) and 7 points in the laboratory domain (persistent high-titer IgG positivity for both anticardiolipin and anti-β2-glycoprotein I antibodies), exceeding the threshold of at least 3 points in each domain. The revised Sydney criteria were likewise fulfilled. Negative antinuclear, anti-double-stranded DNA, extractable nuclear antigen antibodies, normal complement levels, and the absence of clinical features, suggesting systemic lupus erythematosus or another systemic autoimmune disease, supported a designation of primary APS.

Anticoagulation was initiated with heparin bridging followed by warfarin, with a target international normalized ratio of 2.0 to 3.0 and an indefinite intended duration; no concomitant antiplatelet agent was prescribed, given the absence of additional high-risk features. Comprehensive rehabilitation, including physical therapy, was provided, and the modified Rankin Scale score at discharge was 1. Over three months of follow-up, the patient remained free of recurrent vascular events, recurrent chorea, and transient neurological symptoms, with no bleeding complications and no documented difficulty maintaining the target anticoagulation range.

## Discussion

The reference series on APS-associated chorea, which gathered 50 patients from clinical practice and the literature, described features concordant with the present case: a marked female predominance (96%), a mean age at presentation of chorea of 21 ± 12 years, a single episode in most patients (66%), and cerebral infarction on CT or MRI in 35% of cases [5]. Our patient presented with a single episode of unilateral chorea at 18 years of age, with spontaneous resolution within one month, a course phenotypically compatible with previously reported aPL-associated chorea. This phenotypic similarity, however, does not establish that the remote episode was caused by APS.

A potentially relevant aspect is the temporal relationship between chorea and the diagnosis of APS. Chorea has been reported to manifest predominantly before the formal diagnosis of the disease [6], which has led to its consideration as a possible sentinel manifestation. In our patient, 10 years elapsed between the choreic episode and the arterial thrombotic event that allowed classification. Because aPL were not measured at the time of the chorea, it cannot be determined whether the antibodies were already present, and the association remains plausible but unproven.

Patients with childhood- or adolescent-onset APS have been reported to experience choreic episodes more frequently than those with onset at older ages [1], which supports maintaining a high index of suspicion when juvenile-onset chorea occurs without an evident cause.

The pathophysiological mechanism of aPL-associated chorea remains incompletely elucidated, and two main hypotheses have been proposed. The first postulates an ischemic origin due to microthrombosis of the basal ganglia; the second, an immune-mediated dysfunction caused by direct binding of aPL to phospholipid structures within the basal ganglia, with secondary endothelial activation [7]. Functional neuroimaging has documented striatal hypermetabolism in cases of aPL-related chorea studied with 18F-FDG PET, in contrast to the hypometabolism characteristic of neurodegenerative choreas [8], and reversible basal ganglia perfusion abnormalities have been reported on single-photon emission computerized tomography (SPECT) [9]. These observations derive largely from case reports and small series, and they suggest a potentially reversible immune-mediated or functional mechanism in some patients rather than excluding a thrombotic contribution; the two mechanisms may coexist. It should also be noted that spontaneous resolution of symptoms does not by itself establish an immune rather than a vascular mechanism, and the therapeutic implications of metabolic imaging findings remain uncertain.

The differential diagnosis of juvenile-onset chorea must include Sydenham chorea as a priority, given its association with rheumatic fever. This consideration is not trivial: chorea in patients with primary APS has been shown to be significantly associated with a history of rheumatic fever [6], and a systematic review has examined the coexistence of both entities and their potentially shared immunological mechanisms [10]. Other causes that must be excluded include infectious, metabolic, thyroid and drug-related etiologies, particularly oral contraceptive use, described as a trigger of chorea in aPL-positive patients [5], as well as Wilson disease and structural basal ganglia lesions. In our patient, several of these investigations were requested during the initial episode, but the results were not available for review, and she was not taking oral contraceptives at that time.

An important caveat is that the presence of antiphospholipid antibodies in a patient presenting with isolated chorea does not by itself establish clinically significant APS. In a cohort of 18 patients with aPL-associated chorea, only a minority fulfilled criteria for APS, and isolated IgM anticardiolipin positivity predominated among those who did not [11]. Interpretation must therefore be made in the context of the overall clinical picture, including the antibody profile, its persistence, and the presence of other thrombotic or obstetric manifestations.

Management of isolated aPL-associated chorea is not standardized. Symptomatic control has been attempted with dopamine receptor blocking agents, and immunosuppressive therapy with glucocorticoids or other immunomodulatory agents has been used in selected patients, particularly those with associated systemic autoimmune disease [2,11]. The role of antithrombotic therapy is more contentious: in the cohort cited above, anticoagulation was recommended to be reserved for the treatment of thrombosis rather than prescribed for chorea alone in the presence of aPL, since two patients died of bleeding complications [11]. Current evidence therefore does not support routine anticoagulation based on isolated chorea with positive aPL, and management should rest on individualized risk assessment.

In the context of cerebrovascular events, antiphospholipid antibodies have been identified with appreciable frequency among young adults with cerebrovascular events [12], and their presence has been described as an independent risk factor for cerebral ischemia in this population [13]. Rather than advocating universal testing, we would limit the recommendation to patients with cryptogenic or otherwise unexplained stroke, particularly when clinical features suggest APS, systemic autoimmune disease, previous thrombosis, obstetric morbidity, or other non-criteria manifestations. A broader spectrum of APS-associated cerebrovascular lesions has also been described, including white matter hyperintensities, cortical atrophy, and infarcts, with potential neurocognitive sequelae [14].

A distinction should be drawn between clinical diagnosis and fulfillment of classification criteria. The 2023 ACR/EULAR criteria superseded the 2006 revised Sydney criteria for research classification purposes and were not designed to replace clinical judgment; patients may have clinically meaningful APS without fulfilling them. They require as an entry criterion at least one positive aPL test within three years of identification of the associated clinical criterion, followed by an additive weighted system comprising six clinical and two laboratory domains [4]. They prioritize specificity (99%) over sensitivity (84%), which implies that patients with non-criteria manifestations, such as chorea, may fall outside classification despite having clinically active disease [15]. This is particularly relevant in the present case, in which the inaugural manifestation does not score in any clinical domain.

Regarding management of the thrombotic event, anticoagulation with vitamin K antagonists constitutes the treatment of choice in thrombotic APS. The EULAR recommendations for the management of APS in adults advise against the use of direct oral anticoagulants in patients with definite APS and arterial events, given the high risk of thrombotic recurrence, and vitamin K antagonist monotherapy with a target international normalized ratio of 2.0 to 3.0 is considered appropriate for a first arterial event, with the addition of low-dose aspirin or a higher target reserved for selected patients with recurrent events or additional high-risk features [16]. In our patient, monotherapy was chosen after weighing the arterial nature of the event and the double IgG positivity against her young age and absence of bleeding risk factors; no recurrence occurred during follow-up.

This report has several limitations. The most important is that antiphospholipid antibodies were not measured at the time of the choreic episode, so it cannot be established whether they were present or whether the episode was causally related to APS. Information about that episode derives from external medical records in which several investigations are named, but their results are not available, precluding definitive exclusion of alternative etiologies; phenomenological detail is correspondingly limited. Functional neuroimaging was not performed during the choreic episode, and diffusion-weighted and apparent diffusion coefficient images from the acute stroke could not be retrieved, although restricted diffusion is documented in the formal radiological report. Inpatient telemetry and inherited thrombophilia screening were not performed. The laboratory reported assay methodology and positivity cut-offs but not the manufacturer, analytical platform, or laboratory-specific 99th-percentile thresholds, which limits comparability across assays. Follow-up was limited to three months. Finally, this is a single case, which precludes causal inference.

## Conclusions

Otherwise unexplained juvenile-onset chorea may precede thrombotic antiphospholipid syndrome by several years and may represent a possible antecedent non-criteria manifestation. This case provides a clinically relevant illustration of that possibility, while recognizing that the absence of contemporaneous antibody testing leaves the relationship plausible but unproven. Antiphospholipid antibody testing should be considered as part of the etiological evaluation of otherwise unexplained chorea, particularly in young patients or in the presence of other autoimmune or thrombotic features. Earlier identification of persistent high-risk antiphospholipid antibody positivity could permit longitudinal risk assessment, management of modifiable thrombotic risk factors, and individualized consideration of preventive treatment; however, the optimal thromboprophylactic strategy in patients with isolated aPL-associated chorea remains uncertain, and this case cannot demonstrate that any specific preventive therapy would have been indicated or would have averted the subsequent stroke.
